# An Optimization Model for a Wetland Restoration Project under Uncertainty

**DOI:** 10.3390/ijerph15122795

**Published:** 2018-12-10

**Authors:** Baofeng Cai, Yang Zhang, Xianen Wang, Yu Li

**Affiliations:** 1 College of Environment and Resources, Jilin University, Changchun 130012, China; 1357joe@163.com; 2 College of Economics and Management, China Jiliang University, Hangzhou 310018, China; zhy@cjlu.edu.cn; 3 MOE Key Laboratory of Resources and Environmental Systems Optimization, North China Electric Power University, Beijing 102206, China

**Keywords:** wetland restoration project, interval linear programming, wetland management, optimization model, uncertainty

## Abstract

Restoring natural wetlands with conservation projects is an urgent task for human well-being. This paper introduces the Interval linear programming (ILP) method in wetland restoration projects for the first time and builds an optimization model. The purpose of the optimization model is to find an optimal restoration measures allocation pattern that can minimize the total investment in wetland restoration projects and obtain additional ecological environment and socio-economic benefits. The optimization model can also decrease the influence of interval uncertainty in the system by expressing the executed solution as interval numbers with an upper bound and a lower bound. The result of the optimization model for the wetland restoration project indicated a range of 6.84%–15.43% reduction on comparison with the original scheme which verified the effectiveness and validity of this optimization model. Our findings indicate that higher ecological and social benefits of wetland restoration projects can be achieved with lower restoration investment on the application of the reasonable and optimal restoration measures allocation pattern by the optimization model. The results of interval solutions can provide guidance for project managers to select a satisfactory decision-making plan by adjusting the decision variables in the interval solutions according to the practical situation. It can be seen that reeds were suggested to be planted over 46.75 km^2^, with the same lower bound and higher bound. Meanwhile, populus euphratica, and dryland willow were recommended to be planted in a mixed forest pattern within the interval of 30.54 km^2^ to 37.25 km^2^, and so forth. With the optimal solutions obtained from the model, the total project investment would be in the range of 2193.14 (10^4^ CNY) to 2416.01 (10^4^ CNY). Future improvements of our optimization model in wetland restoration projects should consider other kinds of uncertainties in the system such as stochastic uncertainties, fuzzy uncertainties, and integrated uncertainties.

## 1. Introduction

Wetlands are internationally recognized as an indispensable resource for humans, which are distinct ecosystems inundated by water [1,2,3,4,5]. Wetlands represent one of the world’s most important types of ecosystems, as they not only play a critical role in climate change, human health, water resource, and biodiversity, but also provide important ecosystem services such as water quality improvement, flood abatement, and carbon sequestration [6,7,8,9,10,11]. Freshwater wetlands occupy 1% of the global surface water resources but support shelters for more than 40% of the global species [12,13]. However, wetland degradation, especially wetlands salinization, is an indisputable reality, and human activities are chiefly to blame for the main factors. Substantial areas of wetlands have been degraded or have disappeared due to agricultural extension and urban expansion in recent decades. Meanwhile, excessive emissions of wastewater lead to deterioration of the water environment quality and eutrophication, creating further wetland pollution problems [14,15,16,17,18]. Therefore, it is an urgent task to restore natural wetlands with conservation projects for human well-being.

To date, most researches on wetlands restoration focused on restorability assessment [19,20,21,22], suitability prediction [23,24,25,26], restoration site selection [27,28,29,30,31], and so on. Qu et al. (2018) proposed a GIS-based Restorability Index (RESI) model and restoration plan to evaluate the wetland restorability of the Sanjiang Plain, which is the largest marsh area of China [32]. White and Fennessy (2005) developed a GIS-based model to predict the suitability of wetland restoration for all locations in the Cuyahoga River watershed in northeastern Ohio, providing spatially explicit guidance for wetland restoration efforts [33]. Comín et al. (2014) prioritized wetland-restoration sites at the watershed scale as part of a demonstration project for improving wastewater from irrigated agricultural land discharging into the Flumen River [34]. Meanwhile, data for the wetland restoration projects for decision-making has uncertain characteristics, and interrelationships between various parameters can be extremely complicated, such as variability in ecological water requirement, carbon sinks, ecological indicators, and other systematic parameters. In practice, these uncertainties generate enormous challenges for wetland restoration project management. Therefore, effective optimization approaches in wetland restoration project management under these complicated, uncertain conditions are necessary. Unfortunately, no attempts have been made to systematically determine the optimum management plan for wetland restoration projects under uncertainty.

This paper intends to introduce the Interval linear programming (ILP) method in wetland restoration projects for the first time and build an optimization model. The proposed model for wetland restoration projects based on ILP has some advantages over traditional optimization methods in terms of convenient data availability and succinct computational requirements. The purpose of the optimization model is to find an optimal restoration measures allocation pattern that can minimize the total investment in wetland restoration projects and obtain additional ecological environment and socio-economic benefits. The optimization solutions can provide decision-making suggestions for project managers.

## 2. Methods

ILP is a mathematical programming method based on interval theory that can handle interval uncertainty in the objective function and constraint conditions of optimization models. The solution of the ILP model can provide a credible and scientific decision basis for project managers. In this section, we first provide the ILP formulation and its solution algorithm [35,36,37,38,39,40] in a wetland restoration project, and then provide the modelling formulation.

### 2.1. Definitions for Interval Parameter

Prior to formulating the ILP model for wetland restoration projects, we first introduce some ancillary definitions used in previous research, these definitions will be implemented through the solution of the ILP Model to assist with computational efforts.

**Definition 1.**  *Let x denote a closed and bound set of real numbers. An interval number $x^{\pm }$ with a known upper and lower bound but with unknown distribution information is defined as an interval for x such that*

(1) $$x^{\pm }=\left[x^{-},x^{+}\right]=\left\{t\in x|x^{-}\leq t\leq x^{+}\right\}$$

*where $x^{-}$ and $x^{\pm }$ represent the lower and upper bounds of $x^{\pm }$, respectively.*

**Definition 2.**  *For $x^{\pm }$, the following relationships hold:*

(2) $$x^{\pm }\geq 0\;\mathrm{iff}\;x^{-}\geq 0\;\mathrm{and}\;x^{+}\geq 0$$

(3) $$x^{\pm }\leq 0\;\mathrm{iff}\;x^{-}\leq 0\;\mathrm{and}\;x^{+}\leq 0$$

**Definition 3.**  *For* $x^{\pm }=\left[x^{-},x^{+}\right]$ *and* $y^{\pm }=\left[y^{-},y^{+}\right]$ *, the order relationships are as follows:*

(4) $$x^{\pm }\leq y^{\pm }\;\mathrm{iff}\;x^{-}\leq y^{-}\;\mathrm{and}\;x^{+}\leq y^{+}$$

(5) $$x^{\pm }<y^{\pm }\;\mathrm{iff}\;x^{-}<y^{-}\;\mathrm{and}\;x^{+}<y^{+}$$

**Definition 4.**  *For* $x^{\pm }$ *, its absolute value $\left|x^{\pm }\right|$ is defined as follows:*

(6) $$\left|x^{\pm }\right|=\left\{ \begin{array}{l} x^{\pm },\;if\;x^{\pm }\geq 0\\ -x^{\pm },\;if\;x^{\pm }\leq 0 \end{array}\right.$$

*Thus*

(7) $${\left|x\right|}^{+}=\left\{ \begin{array}{l} x^{+},\;if\;x^{\pm }\geq 0\\ -x^{-},\;if\;x^{\pm }<0 \end{array}\right.$$

*and*

(8) $${\left|x\right|}^{-}=\left\{ \begin{array}{l} x^{-},\;if\;x^{\pm }\geq 0\\ -x^{+},\;if\;x^{\pm }<0 \end{array}\right.$$

**Definition 5.**  *Let* ∗∈{+,−,×,÷} *be a binary operation on interval numbers. For* $x^{\pm }$ *and $y^{\pm }$:*

(9) $$x^{\pm }*y^{\pm }=\left[\min\left\{x*y\right\}|,\max\left\{x*y\right\}\right],\;x^{-}\leq x\leq x^{+},y^{-}\leq y\leq y^{+}$$

### 2.2. Interval Linear Programs

Let denote a set of interval numbers. An ILP model can be defined as follows:

(10a) $$Max\;f^{\pm }=C^{\pm }X^{\pm }$$

subject to:

(10b) $$A^{\pm }X^{\pm }\geq B^{\pm }$$

(10c) $$X^{\pm }\geq 0$$

where $A^{\pm }\in {\left\{R^{\pm }\right\}}^{m\times n},B^{\pm }\in {\left\{R^{\pm }\right\}}^{m\times 1},C^{\pm }\in {\left\{R^{\pm }\right\}}^{1\times n},X^{\pm }\in {\left\{R^{\pm }\right\}}^{n\times 1}$.

### 2.3. Solution of the ILP Model

The ILP formulation used in this paper was borrowed and its solution algorithm was developed by Huang et al. [41] to solve the problem in waste management. This type of solution may be favored by decision-makers because of its flexibility. As our method does not lead to complicated intermediate models, it also has reasonable computational requirements. According to the algorithm, model (1) can be solved through a two-step process. The first step is to formulate a sub-model corresponding to *f*^+^ and solve it by maximizing the objective. The second step is to solve a sub-model corresponding to *f*^−^, based on the upper bound solution generated in the first step.

The sub-model corresponding to *f*^+^ is formulated as follows (assuming that $b_{i}^{\pm }\geq 0$):

(11a) $$Max\;f^{+}=\sum_{j=1}^{k_{1}}c_{j}^{+}x_{j}^{+}+\sum_{j=k_{1}+1}^{n}c_{j}^{+}x_{j}^{-}$$

subject to:

(11b) $$\sum_{j=1}^{k_{1}}{\left|a_{ij}\right|}^{-}sign\left(a_{ij}^{-}\right)x_{j}^{+}+\sum_{j=k_{1}+1}^{n}{\left|a_{ij}\right|}^{+}sign\left(a_{ij}^{+}\right)x_{j}^{-}\leq b_{i}^{+},\forall i$$

(11c) $$x_{j}^{+}\geq 0,x_{j}^{-}\geq 0,x_{j}^{\pm }\in X^{\pm },j=1,2,\ldots n$$

(11d) $$c_{j}^{\pm }\geq 0,j=1,2,\ldots k_{1}$$

(11e) $$c_{j}^{\pm }\leq 0,\;j=k_{1}+1,k_{1}+2,\ldots n$$

Solutions of $x_{j\;opt}^{+}$ ($j=1,2,\ldots k_{1}$), $x_{j\;opt}^{-}$ ($j=k_{1}+1,k_{1}+2,\ldots n$), and $f_{opt}^{+}$ can be obtained using sub-model (2). The sub-model corresponding to *f*^−^ can then be formulated as follows:

(12a) $$Max\;f^{-}=\sum_{j=1}^{k_{1}}c_{j}^{-}x_{j}^{-}+\sum_{j=k_{1}+1}^{n}c_{j}^{-}x_{j}^{+}$$

subject to:

(12b) $$\sum_{j=1}^{k_{1}}{\left|a_{ij}\right|}^{+}sign\left(a_{ij}^{+}\right)x_{j}^{-}+\sum_{j=k_{1}+1}^{n}{\left|a_{ij}\right|}^{-}sign\left(a_{ij}^{-}\right)x_{j}^{+}\leq b_{i}^{-},\forall i$$

(12c) $$x_{j}^{-}\leq x_{j\;opt}^{+},j=1,2,\ldots k_{1}$$

(12d) $$x_{j}^{+}\geq x_{j\;opt}^{-},j=k_{1}+1,k_{1}+2,\ldots n$$

(12e) $$x_{j}^{-}\geq 0,\;x_{j}^{+}\geq 0,\;x_{j}^{\pm }\in X^{\pm },\;j=1,2,\ldots ,n$$

Solutions of $x_{j\;opt}^{-}$ ($j=1,2,\ldots k_{1}$), $x_{j\;opt}^{+}$ ($j=k_{1}+1,k_{1}+2,\ldots n$), …, *k*_1_), and $f_{opt}^{-}$ can be obtained using sub-model (3). Thus, the general solutions can be obtained as follows:

(13a) $$f_{opt}^{\pm }=[f_{opt}^{-},f_{opt}^{+}]$$

(13b) $$x_{j\;opt}^{\pm }=[x_{j\;opt}^{-},x_{j\;opt}^{+}],\;\forall j$$

If the objective function is to be minimized, then the sub-model corresponding to *f*^−^ should be solved first.

### 2.4. Wetland Restoration Optimization Model

To decrease the influence of uncertainty from system parameters and their interrelationships on the executed solution in the wetland restoration optimization model, all variables in the optimization model were expressed as interval numbers with an upper bound and a lower bound. The objective of the wetland restoration optimization model is to minimize the total investment of the wetland restoration project subject to the decision variables and the relationships between the decision variables and the objective. A complete list of decision variables is provided after the model.

The optimization model can be formulated as follows:

(14a) $$Minf^{\pm }=\sum_{i=1}^{I}Q_{i}^{\pm }\cdot L_{i}^{\pm }\cdot x_{i}^{\pm }$$

where *f*^±^ is the total expected system benefit (10^4^ Chinese Yuan (CNY)) over the planning periods; *i* denotes the *i*th restoration measures; $Q_{i}^{\pm }$ represents seedling price per *i*th restoration measures (10^4^ CNY/plant); $L_{i}^{\pm }$ represents the planting density in the *i*th restoration measures (plant/km^2^); $x_{i}^{\pm }$ represents the areas of *i*th restoration measures (km^2^).

Constraints:

(1) Ecological water demand constraints:

(14b) $$\sum_{i=1}^{I}\left(W_{l}^{\pm }+m_{i}^{\pm }x_{i}^{\pm }+W_{m}^{\pm }+W_{s}^{\pm }+W_{a}^{\pm }\right)\leq W_{0}^{\pm }$$

where $m_{i}^{\pm }$ represents the ecological water demand quota of the *i*th restoration measures (10^4^ m^3^/km^2^); $W_{l}^{\pm }$ represents the water requirement of the lake (10^4^ m^3^); $W_{m}^{\pm }$ represents the water requirement of the marsh (10^4^ m^3^); $W_{s}^{\pm }$ represents the water requirement of the soil (10^4^ m^3^); $W_{a}^{\pm }$ represents the water requirement of the wildlife habitat (10^4^ m^3^); $W_{o}^{\pm }$ represents the maximum total water requirement in the wetland (10^4^ m^3^).

(2) Salinization constraints:

(14c) $$\sum_{i=1}^{1}h_{i}^{\pm }\cdot c_{i}^{\pm }\cdot M\cdot \omega_{i}^{\pm }\cdot x_{i}^{\pm }\geq N_{0}^{\pm }$$

where $h_{i}^{\pm }$ represents the soil thickness in the *i*th restoration measures (m); $c_{i}^{\pm }$ represents the chloride concentration in the *i*th restoration measures (mol/m^3^); *M* represents the molecular weight of chloride (g/mol); $\omega_{i}^{\pm }$ represents the chloride absorption efficiency of the *i*th restoration measures; $N_{0}^{\pm }$ represents the total absorption of chloride during the project implementation period (tonnes).

(3) Carbon sink constraints:

(14d) $$\sum_{i=1}^{I}C_{i}^{\pm }\cdot x_{i}^{\pm }\geq C_{o}^{\pm }$$

where $C_{i}^{\pm }$ represent capacity per unit area of vegetation type in the *i*th restoration measures (tonnes/km^2^); $C_{o}^{\pm }$ represents the total carbon sink capacity (tonnes).

(4) Total area constraints:

(14e) $$\sum_{i=1}^{I}x_{i}^{\pm }\leq A_{0}^{\pm }$$

where $A_{o}^{\pm }$ represents the total area of the wetland restoration project (km^2^).

(5) Labor force constraints:

(14f) $$\sum_{i=1}^{I}L_{ki}^{\pm }\cdot x_{i}^{\pm }\leq L_{0}^{\pm }$$

where $L_{ki}^{\pm }$ represents the labor coefficient per unit area of vegetation type in the *i*th restoration measures for meeting local production needs (man-day/ha); $L_{0}^{\pm }$ represents gross labor force (man-day).

(6) Ecological benefit constraints:

(14g) $$V_{A}^{\pm }+V_{W}^{\pm }+V_{C}^{\pm }\geq V_{0}^{\pm }$$

(14h) $$V_{A}^{\pm }=\sum_{i=1}^{I}P_{Ai}^{\pm }\cdot \beta_{i}^{\pm }\cdot x_{i}^{\pm }$$

(14i) $$V_{W}^{\pm }=\sum_{i=1}^{I}P_{Wi}^{\pm }\cdot \delta_{i}^{\pm }\cdot x_{i}^{\pm }$$

(14j) $$V_{C}^{\pm }=P_{Ci}^{\pm }\cdot \varepsilon_{i}^{\pm }\cdot x_{i}^{\pm }$$

where $V_{A}^{\pm }$ represents the benefits of microclimate regulation; $V_{W}^{\pm }$ represents the benefits of water purification; $V_{C}^{\pm }$ represents the benefits of soil and water conservation; $V_{0}^{\pm }$ represents the total ecological benefits; $P_{Ai}^{\pm }$ represents the savings in electricity consumption due to temperature regulation by the *i*th wetland restoration measures (10^4^ CNY/km^2^); $\beta_{i}^{\pm }$ represents the correction coefficient of temperature regulation by the *i*th wetland restoration measures; $P_{Wi}^{\pm }$ represents the savings in sewage treatment cost due to the *i*th wetland restoration measures (10^4^ CNY/km^2^); $\delta_{i}^{\pm }$ represents the correction coefficient of water purification by the *i*th wetland restoration measures; $P_{Ci}^{\pm }$ represents the savings in cost of soil and water conservation due to the *i*th wetland restoration measures (10^4^ CNY/km^2^); $\varepsilon_{i}^{\pm }$ represents the correction coefficient of soil and water conservation by the *i*th wetland restoration measures.

(7) Social benefit constraints:

(14k) $$\sum_{i=1}^{I}\alpha_{i}^{\pm }\cdot U_{i}^{\pm }\cdot x_{i}^{\pm }\geq U_{0}^{\pm }$$

where $U_{i}^{\pm }$ represents the economic benefit of plants in the *i*th wetland restoration measures (10^4^ CNY/tonnes) $\alpha_{i}^{\pm }$ represents the yield per unit area of economic plants in the *i*th wetland restoration measures (tonnes/km^2^); $U_{0}^{\pm }$ represents the total economic benefit during the project implementation period (10^4^ CNY).

(8) Other constraints:

(14l) $$x_{i}^{\pm }\geq 0$$

## 3. Case Study

**Figure 1:**
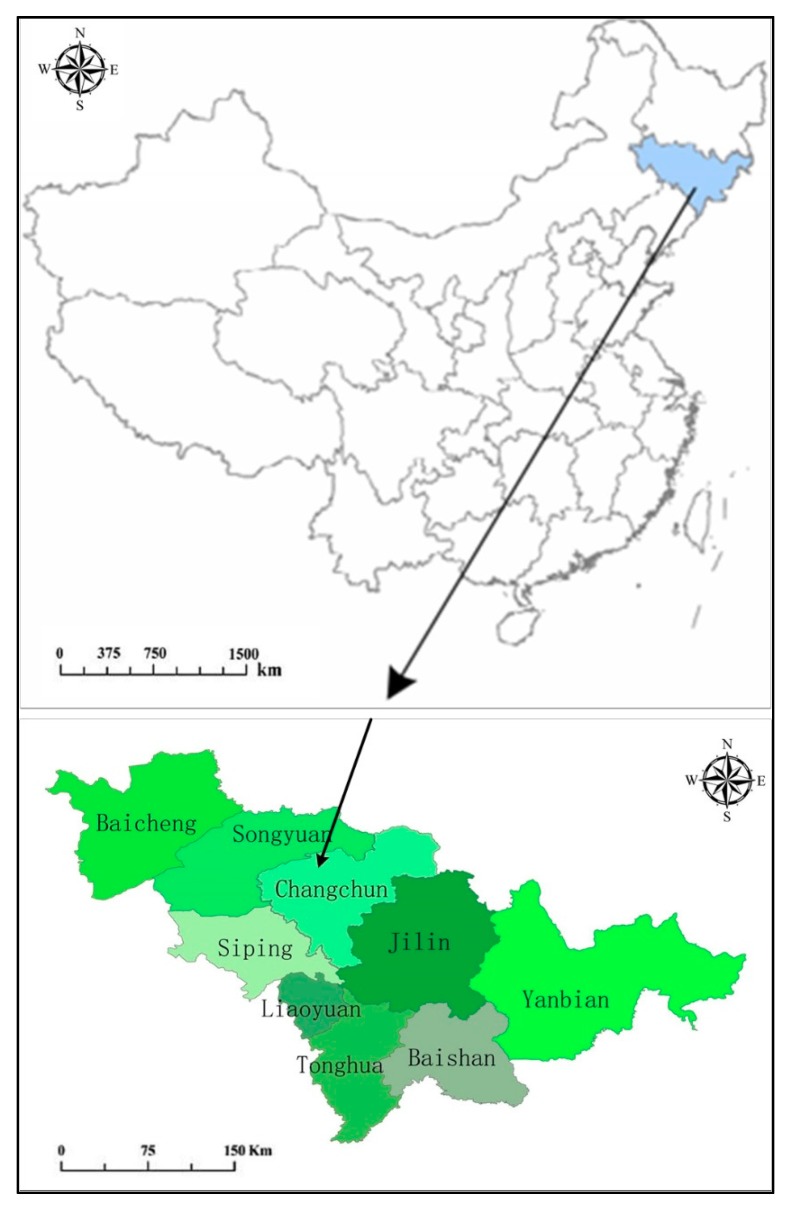
Map of the wetland restoration project (shallow basket lake wetland) in Jilin province, China.

The shallow basket lake wetland is located in the west part of Nongan County, Changchun City, Jilin Province, which is 35 km away from Nongan County and 60 km away from Changchun City. It is adjacent to five counties. It is the biggest inland alkaline freshwater lake in Changchun City and the sole natural wetland in Jilin Province. The total wetland area is 280 km^2^ and the surface water area is 100 km^2^. The water depth ranges from 1 m to 2.5 m. The length of the surface water is 25 km from north to south and 10 km from east to west. It is in the north temperature continental monsoon climate zone. The mean annual temperature is 4.9 °C with 35.8 °C the highest temperature and −36.1 °C the lowest temperature. The daily annual solar radiation is 14.87 MJ/m^2^·d and the sunshine duration index is 2630–2930. The relative humidity ranges from 52% to 61%. The map of the project area is shown in Figure 1.

The shallow basket lake wetland has a flow cutoff in some rivers in terms of long-term drought and shortage of rain. The plummeting groundwater table also led to a decrease in wetland and grass area, and an increase in groundwater recession, soil erosion, soil desertification, and salinization, which had seriously threatened ecological security. The total salinized area in the shallow basket lake wetland is 3650.04 km^2^ with 1800.59 km^2^ of slight salinized area (49.33%), 833.82 km^2^ of medium salinized area (22.84%), and 1015.63 km^2^ of high salinized area (27.83%).

Different vegetation types will be selected as the restoration measures for the wetland restoration project. To maintain the health of the wetland and satisfy the project objectives, the project will select a suitable amount of reed and tree species to prevent wetland salinization and to increase the carbon sink. Each tree species will be determined considering local resources. The selected tree species and the areas of the recommended scheme are shown in Table 1. Socioeconomic data used in this paper were obtained from the China City Statistical Yearbooks and National Economy [42], and Society Developed Statistical Bulletins [43] which are published by the National Bureau of Statistics of the People’s Republic of China and Changchun Municipal Bureau of Statistics, respectively.

## 4. Results and Discussion

Table 2 presents the optimal solutions of wetland restoration measures on solving model (5) by the ILP method. It can be seen that reeds were suggested to be planted over 46.75 km^2^, with the same lower bound and higher bound. Meanwhile, populus euphratica, and dryland willow were recommended to be planted in mixed forest pattern within the interval from 30.54 km^2^ to 37.25 km^2^, and so forth. With the optimal solutions obtained from the model, the total project investment would be in the range from 2193.14 (10^4^ CNY) to 2416.01 (10^4^ CNY). Note that the solutions of the objective function value and decision variables are interval. Generally, the interval solutions of the objective function value and decision variables are presented with upper bound and lower bound. These interval results of the wetland restoration optimization model indicate that the final decisions are sensitive to uncertain inputs for project managers. In contrast, certain solutions from the wetland restoration optimization model with the traditional method are not sensitive to the input uncertainties. Thus, alternative schemes of the wetland restoration optimization project can be achieved by adjusting the interval solutions in the range of its lower and upper bounds according to the various project management requirements. For example, the solutions of $x_{2}^{\pm }$ under the given constraints reflect intervals of planting area for populus euphratica and dryland willow with mixed forest planting pattern. The upper bound of $x_{2}^{\pm }$ (i.e., $x_{2}^{+}$) corresponds to a higher objective result and the lower bound of $x_{2}^{\pm }$ (i.e., $x_{2}^{-}$) corresponds to a lower objective result.

**Figure 2:**
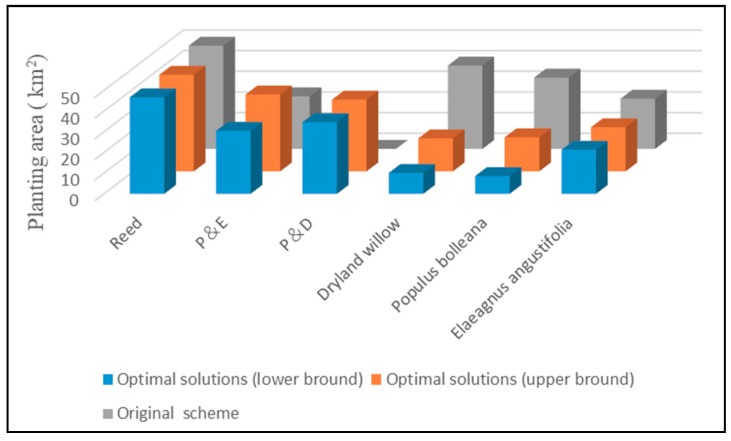
The wetland restoration measures between the original scheme and optimal solutions.

The solution in the wetland restoration project was $f_{opt}^{\pm }$ = [2193.14, 2416.01] (10^4^ CNY) which provided the interval range of total wetland restoration project investment under the optimal restoration measures allocation pattern. As the actual value of each system variable or input parameter could be any value in its interval, the total investment of the wetland restoration project would change between $f_{opt}^{-}$ and $f_{opt}^{+}$ as the system variables changed. A restoration measures allocation pattern with lower-bound leads to a lower project investment. Conversely, a restoration measures allocation pattern with upper-bound leads to a higher project investment. Therefore, the optimal solutions obtained from the model are flexible in reflecting possible system condition variations in terms of the existence of input uncertainties. According to the feasibility design report, the total investment of projects in the original scheme was 2593.41 (10^4^ CNY). Compared with the project’s original scheme, 177.4–400.27 (10^4^ CNY) of the project investment can be saved if the optimal restoration measures obtained from the model are implemented by the project managers. The result of the optimization model for wetland restoration project indicated a range of 6.84%–15.43% reduction which verified the effectiveness and validity of this optimization model. Figure 2 presents the original scheme and optimal solutions of wetland restoration measures.

**Figure 3:**
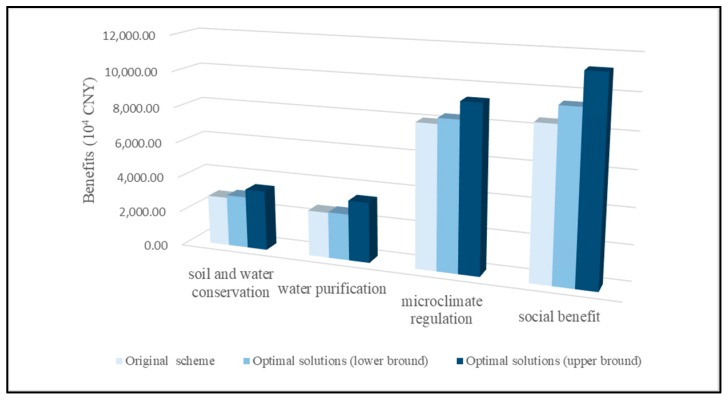
The ecological and social benefits for the recommended scheme and the optimal solutions.

The ecological and social benefits of wetland restoration project in relative terms with original scheme and optimal solutions were different. Although the total investment of the wetland restoration project obtained from the optimal solutions was lower than that from the original scheme, the ecological and social benefits of the wetland restoration project obtained from the optimal solutions are higher than that from original scheme. In the optimal solutions, the ecological and social benefits also increased with increasing restoration investment. The benefits of soil and water conservation, water purification, microclimate regulation, and society in the original scheme were 2804.74, 2589.93, 8039.90, and 8521.81 (10^4^ CNY), respectively. The four kinds of ecological and social benefits obtained from the optimal solutions by the optimization model were [2,655, 3,067], [2,357, 3,064], [7,516, 8,382] and [8,515, 10,133] (10^4^ CNY) respectively. Figure 3 compares these benefits under the recommended scheme and the optimal solutions. Comparing the benefits of the original scheme, there was correspondingly 5.26%, 1.20%, 3.95%, and 11.11% increase with the lower bound of the optimal solutions. For the difference between the original scheme and the upper bound of the optimal solutions, the four kinds of ecological and social benefits showed 21.60%, 31.55%, 15.93%, and 32.22% of growth. In conclusion, higher ecological and social benefits of wetland restoration projects can be achieved with lower restoration investment with the application of the reasonable and optimal restoration measures allocation pattern by the optimization model. This conclusion is also consistent with the results from Li et al. (2013) and Zhang et al. (2016), which indicated that a scientific wetland restoration scheme can lead to optimal ecological benefits (e.g., soil and water conservation, water purification, microclimate regulation etc.) with lower project investment [44,45].

This optimization model for the wetland restoration project based on ILP has some advantages over traditional optimization methods in terms of convenient data availability and succinct computational requirements. It can effectively manage uncertainties existing in system variables or input parameters and achieve robust and feasible interval solutions. The results of our case study show that decision-makers can not only identify the optimal restoration measures allocation pattern, but also minimize the total investment of the wetland restoration project while obtaining additional ecological environment and socio-economic benefits. When the fluctuation of system variables or input parameters with uncertain characteristics changed in their interval ranges, the total investment of the wetland restoration project also changed between the range of 2193.14–2416.01 (10^4^ CNY). Therefore, the project managers can obtain a satisfactory decision-making plan by adjusting the decision variables in the interval solutions according to the practical situation. However, there are some limitations in the proposed model. The constructed optimization model for a wetland restoration project can only handle interval uncertainties in the system but no stochastic, fuzzy or other kinds of uncertainties. In addition, this model does not consider the decision risk under uncertainty in this study. 

## 5. Conclusions

In conclusion, this paper introduced the ILP method in wetland restoration projects for the first time and built an optimization model to find an optimal restoration measures allocation pattern that can minimize the total investment in wetland restoration projects and obtain additional ecological environment and socio-economic benefits. This model can decrease the influence of uncertainty from system parameters and their interrelationships on the executed solution in the wetland restoration optimization model by expressing as interval numbers with an upper bound and a lower bound.

The result of the optimization model for the wetland restoration project indicated a range of 6.84%–15.43% reduction on comparison with the original scheme which verified the effectiveness and validity of this optimization model. It can be seen that reeds were suggested to be planted over 46.75 km^2^, with the same lower bound and higher bound. Meanwhile, populus euphratica, and dryland willow were recommended to be planted in a mixed forest pattern within the interval from 30.54 km^2^ to 37.25 km^2^, and so forth. With the optimal solutions obtained from the model, the total project investment would be in the range of 2193.14 (10^4^ CNY) to 2416.01 (10^4^ CNY). Furthermore, the ecological and social benefits of the wetland restoration project in relative terms of the original scheme and optimal solutions were different. Although the total investment of the wetland restoration project obtained from the optimal solutions were lower than that from the original scheme, the ecological and social benefits of the wetland restoration project obtained from the optimal solutions are higher than that from the original scheme. It can be seen that higher ecological and social benefits of the wetland restoration project can be achieved with lower restoration investment on the application of the reasonable and optimal restoration measures allocation pattern by the optimization model. Therefore, the project managers can obtain a satisfactory decision-making plan by adjusting the decision variables in the interval solutions according to the practical situation.

This study can be replicated at different spatial and temporal scales using context-specific datasets to directly support project managers with decision-making on the optimal restoration scheme. Future improvements in our optimization model in wetland restoration projects should consider other kinds of uncertainties in the system such as stochastic uncertainties, fuzzy uncertainties, and integrated uncertainties.

## Figures and Tables

**Table 1:** The recommended scheme for the wetland restoration project.

Restoration Measures	Planting Pattern	Planting Area (km^2^)
Reed (Phragmites karka)	-	50
Populus euphratica, Dryland willow	Mixed forest	25.45
Populus bolleana	Pure forest	34.55
Dryland willow	Pure forest	40.37
Elaeagnus angustifolia	Pure forest	24.29

**Table 2:** Optimal solutions obtained from the model.

Restoration Measures	Planting Pattern	Symbol	Planting Area (km^2^)
Reed (Phragmites karka)	-	$x_{1}^{\pm }$	46.75
Populus euphratica, Dryland willow (P&D)	Mixed forest	$x_{2}^{\pm }$	[30.54, 37.25]
Populus euphratica, Elaeagnus angustifolia (P&E)	Mixed forest	$x_{3}^{\pm }$	34.71
Dryland willow	Pure forest	$x_{4}^{\pm }$	[10.16, 15.97]
Populus bolleana	Pure forest	$x_{5}^{\pm }$	[8.64, 16.48]
Elaeagnus angustifolia	Pure forest	$x_{6}^{\pm }$	21.36
Total project investment (10^4^ CNY): $f_{opt}^{\pm }$ = [2193.14, 2416.01]			
